# Mitochondrial Complex I Deficiency among Egyptian Pediatric Patients with Steroid-Resistant Nephrotic Syndrome

**DOI:** 10.1155/2021/6645373

**Published:** 2021-05-18

**Authors:** Doaa M. Abdou, AbdelAal Mohamed, Mohamed Abdulhay, Sara El Khateeb

**Affiliations:** ^1^Clinical and Chemical Pathology, Faculty of Medicine, Cairo University, Cairo 11562, Egypt; ^2^Internal Medicine Department, Faculty of Medicine, Cairo University, Cairo 11562, Egypt; ^3^Pediatric Department, Faculty of Medicine, Helwan University, Cairo 11795, Egypt

## Abstract

**Results:**

Positive consanguinity was a remarkable finding in 44 patients among the SRNS group (73%), compared with 33 patients among the SSNS group (55%). Complex I activity was significantly lower in the SRNS group (0.2657 ± 0.1831 nmol/ml/min), than in the SSNS group (0.4773 ± 0.1290 nmol/ml/min) (*p* < 0.001). There was a significant positive correlation between complex I activity and the heaviness of proteinuria among the SRNS group (*r* 0.344, *p* < 0.001). There were statistically significant differences in serum C3 and C4 levels between both groups (*p* < 0.001, 0.053, respectively).

**Conclusion:**

Mitochondrial complex I deficiency in patients who have a nephrotic syndrome complaint may play a role in their responsiveness to steroid therapy and the development of SRNS and even the prognosis of their illness.

## 1. Introduction

Nephrotic syndrome (NS) is a clinical condition characterized by massive proteinuria (>3 gm urinary proteins), hypoalbuminemia, edema, and hyperlipidemia [1]. It is one of the most common glomerular diseases among children, with an average incidence of NS of 2–16.9 per 100,000 children worldwide [2]. Approximately 80% of children achieve complete remission within 4 weeks of corticosteroid therapy and are considered steroid-sensitive nephrotic syndrome (SSNS) [3]. Most of steroid-responsive patients (60–70%) will experience >1 relapse after complete remission, 30% of whom will be classified as steroid dependent [4]. The third subclass is steroid-resistant nephrotic syndrome (SRNS) defined as nonremission despite 4 weeks of daily prednisone therapy at a dose of 2 mg/kg/d [3]. SRNS generally, and focal segmental glomerulosclerosis (FSGS) in particular, is associated with a 50% risk for end-stage renal disease (ESRD) within 5 years of diagnosis if patients do not achieve a partial or complete remission [5].

Mitochondrial disease is a heterogeneous group of disorders with the most dominant clinical manifestations associated with neurological and muscular phenotypes; however, with increased awareness and recognition of mitochondrial disease, the spectrum of clinical manifestations has expanded to include other organ systems, such as the heart, liver, gastrointestinal tract, and kidney [6].

Renal manifestations may be a part of well-recognized mitochondrial disease syndromes, such as MELAS (mitochondrial encephalomyopathy, lactic acidosis, and stroke-like symptoms), MERRF (myoclonus, epilepsy with ragged red fibers), Pearson syndrome, Kearns–Sayre syndrome, and Leigh syndrome or as the only disease manifestation or with clinical features that do not fit neatly into a defined syndrome [7].

Deficiency of the mitochondrial respiratory chain enzyme complex I (NADH: ubiquinone oxidoreductase) is considered to be the most frequently encountered single enzyme deficiency in mitochondrial disorders [8]. Both the structural building blocks and the additional protein subunits of Complex I are encoded by 44 different genes [9]. Pathogenic mutations in these genes have been detected, providing the genetic basis for the disease of these patients, and can be considered to be primary complex I defects [10].

The objective of the current study was to detect the frequency of mitochondrial complex I deficiency among Egyptian pediatric patients diagnosed with SRNS and its role in the prognosis for further molecular study of the underlying genetic disorder.

## 2. Materials and Methods

This study was conducted at the inherited metabolic diseases laboratory, Cairo University Children's hospital (CUCH), Faculty of Medicine, Egypt. 120 pediatric patients were included in this study after clinical recruitment on the bases of clinical and laboratory criteria for the diagnosis of nephrotic syndrome. The patients were further divided into two groups, 60 patients who responded to steroid therapy (SSNS) as a control group and 60 patients who did not respond to steroid therapy (SRNS). This study was approved by the Cairo University research ethical committee (REC) (reference no. N-15-2018). Parents of patients and controls gave written informed consent to all the procedures performed in the study.

120 Egyptian pediatric patients, 67 male (55.8%) and 53 females (44.2%), were included; regarding the SRNS group, the mean age of patients was 7.5 ± 3 years, while among the SSNS, it was 6.4 ± 2.9 years. They were recruited from the pediatric nephrology outpatient clinic, Faculty of Medicine, Cairo University. They were diagnosed as NS according to the clinical and laboratory criteria, 60 patients (50%) were suspected of having SRNS due to lack of remission after steroid therapy for 4 weeks, and 60 patients were diagnosed as SSNS after steroid therapy. All patients underwent renal biopsy using a 14-gauge biopsy needle to give two cores, one for the pathological study and the other one was frozen in liquid nitrogen and kept at−80 till biochemical complex I assay was performed (all demographic, clinical, pathological, and biochemical data are available in the supplementary Table).

Samples were homogenized on ice, 1 : 9 (*w*/*v*), in 320 mmol/L sucrose, 1 mmol/L ethylene diamine tetra acetic acid (EDTA), and 10 mmol/L Trizma-base, pH 7.4, using a prechilled hand-held glass homogenizer. Approximately 15–20 mg of renal tissue was homogenized per sample within 9–10 cycles and then transferred to Eppendorf tubes.

Mitochondrial respiratory chain enzyme and citrate synthase (CS) activities were determined by spectrophotometric enzyme assay [11, 12]. Complex I (NADH: ubiquinone reductase, EC1.6.5.3) activity was measured by the rotenone-sensitive oxidation of NADH at 340 nm, while CS (EC 2.3.3.1) activity was determined by the formation of 5-thio-2-nitrobenzoic acid following the incubation of tissue homogenate with acetyl-CoA, oxaloacetate, and 5,50-dithiobis-(2-nitrobenzoic acid), at 412 nm [12]. Complex I activity was expressed as a ratio to the activity of CS, a mitochondrial marker enzyme, to standardize the mitochondrial enrichment of the sample in nmol/ml/min. The range, mean, and standard deviation and Z-score were calculated. Statistical significance between patients and control groups were expressed as the p value (significance considered <0.001).

## 3. Results

This study included 120 patients divided into two groups, group I represented SRNS (60 patients) and group II represented the SSNS (60 patients). There was a statistical significant difference between both groups including 53 females (44.2%) and 76 males (55.8%). There was a statistically significant difference of the age at the time of diagnosis between the SRNS group (Mean ± SD 7 ± SD years) compared with the SSNS (Mean ± SD 6.5 ± 2.8 years) (*p* < 0.001).

The main pathological findings were focal segmental glomerulosclerosis (FSGS) in 44 (73.3%) patients of SRNS, and 40 patients of SSNS (66.7%), followed by minimal changes glomerulonephritis (MCGN) with 8 patients (13.3%) in group I and 16 patients (26.6%) in group II; meanwhile, nonspecific pathological findings were detected in 7 patients (11.7%) of group I, and 4 patients (6.7%) of group II. IgA nephropathy was a pathological finding in only one patient in group I (1.7%), but was not a finding among group II.

Proteinuria is a crucial laboratory finding for diagnosis of nephrotic syndrome; among the patients included in this study, the 24 hrs urinary protein level was 4.5868 ± 1.2451 g/day in the SRNS group, while it was 3.7205 ± 1.466 g/day in the SSNS group (*p*=0.001).

There was a statistically significant difference in serum C3 concentration between both groups; it was 77.23 ± 21.4 mg/dl among the SRNS group, but was 95.78 ± 32 mg/dl among the SSNS group (*p* 0.000). There was a significant difference between both groups regarding serum C4 concentration (0.053); its median level was 7.4 mg/dl (25^th^ 4.5–75^th^ 44.5) in group I, while its median level was 5.5 mg/dl in group II (25^th^ 2.7–75^th^ 10.2).

**Figure 1:**
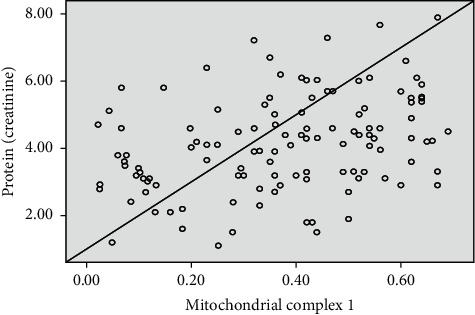
Correlation regression analysis between complex I activity and severity of proteinuria among the studied groups.

Complex I assay was performed in all renal biopsies for all patients; the activity was 0.2657 ± 0.1831 nmol/ml/min among the SRNS group; on the other hand, it was 0.4773 ± 0.1290 nmol/ml/min among the SSNS group with a statistically significant difference between both groups (*p* < 0.001). There was a statistical positive correlation between complex I activities in renal biopsies of SRNS patients and the severity of proteinuria (*r* 0.344, *p* 0.00) (Figure 1).

## 4. Discussion

Nephrotic syndrome is a common glomerular disease in children worldwide, with significant variability in both its incidence and different steroid responsiveness among various ethnic groups [13]. The average incidence of nephrotic syndrome is 2–16.9 per 100,000 children. Understanding the variability by ethnicity may point to potential factors leading to nephrotic syndrome, which remains elusive, and may highlight factors accounting for differences in medication response [2].

Mitochondrial respiratory chain enzyme NADH-Q oxidoreductase (complex I) is coded by a mixture of nuclear genes and mitochondrial genes [14]. Most disorders of mitochondrial complexes in children are caused by mutations in nuclear genes, although mutations in mitochondrial DNA have also been found [9]. A problem with mitochondrial respiratory chain enzymes disorders is that mutations may only be expressed in affected tissues and, sometimes, genetic screening does not give a definite diagnosis, so further biochemical and immunohistochemically investigations may be required [12]. Consequently, assessment of the affected organ may be more appropriate in spite of invasiveness, but confirmation of abnormal respiratory chain enzymes may then be helpful in determining the most appropriate genetic tests [14] using CS activity as a reference controlled for variables such as the mitochondrial enrichment of the sample [11].

This study has illustrated the possibility of measuring mitochondrial complex I in renal tissue obtained by the routine clinical procedure of renal biopsy and to outline its role in development and the prognosis of SRNS. This is the first time to measure complex I in renal tissue. This is important because it indicates that age-specific reference intervals may not be necessary for renal complex I activity if is expressed as a ratio to CS activity.

120 children were included in this study; their median age was 7 years, ranging from 1.5 years to 15 years. The study included 53 females (44.2%) and 67 males (55.8%). Positive consanguinity was recorded in 44 patients of the SRNS group (73%) and 33 patients of the SSNS group (55%).

Among the patients diagnosed as SRNS, the range of complex I activity was 0.022 to 0.64 (Mean 0.2657, SD 0.1831) compared with 0.21 to 0.66 among the SSNS (Mean 0.4773, SD 0.1290). According to our results, the range of complex I activity in renal tissue was different than its corresponding in both skeletal and liver tissues (0.104–0.268; 0.054–0.22 nmol/ml/min, respectively), but in accordance with the work of Ghose et al. [14, 15] who reported the same findings, in spite of the variability of complex I activity ranges compared to our study [12]. This can be explained by the ethnic variations and sample size difference.

Mitochondrial diseases have revealed dramatic variability in the phenotypic presentation; meanwhile, phenotypic manifestation of the genetic defect occurs only when a threshold level is exceeded, and this phenomenon has been named the “phenotypic threshold,” so follow-up is mandatory for the remaining highly suspected patients who had no complex I deficiency [16].

The current study demonstrated that there was a statistically significant difference in complex I activity among the SRNS group than in the SSNS group (*p* < 0.001). The mean activity, standard deviation (SD), and Z-score from all the samples' activities were calculated, and accordingly, complex I deficiency was verified in 25/60 patients among the SRNS group (41.6%) where 17/25 had FSGS, 4/25 had MCGN, and 3/25 had nonspecific pathological findings. Regarding the consanguinity, 19/25 were with positive consanguinity and 6/25 were with negative consanguinity, a finding that enforces the concern of autosomal recessive mode of inheritance, for further molecular work up for the most common gene variants or whole exome sequencing.

5/60 patients among the SSNS group (8.3%), with 4/5 patients showing FSGS, and 1/5 patients had minimal change disease in their pathological study. All patients had positive consanguinity.

According to our results, the range of complex I activity in renal tissue was higher than its corresponding one in both skeletal and liver tissues (0.104–0.268; 0.054–0.22 nmol/ml/min respectively); this is in accordance with the work of Ghose et al. [14, 15] who reported the same findings, in spite of the variability of complex I activity ranges than our study [12]. This can be explained by the ethnic variations and sample size difference.

To the best of our knowledge this is the first study in Egypt for measuring mitochondrial complex I assay in renal biopsies, despite of the possibility of high prevalence of autosomal recessive mitochondrial disorders due to the high rate of positive consanguinity marriage.

The shortage of the current study was the inability to determine whether tissues from the renal medulla or cortex have different complex I activities, which can be overcome by renal biopsies from different sites for measuring complex I and to assess its activity variability between them.

## 5. Conclusions

Mitochondrial complex I deficiency may play a role in the pathogenesis of nephrotic syndrome and the responsiveness to steroid therapy.

## Data Availability

Data are available upon request.

## Supplementary Materials

Supplementary Materials Demographic, clinical, pathological, and biochemical data are available in the supplementary table.
