# Supplementary material for: Mitochondrial Complex I Deficiency among Egyptian Pediatric Patients with Steroid-Resistant Nephrotic Syndrome
Source: Int J Nephrol. 2021 May 18;2021:6645373. doi: 10.1155/2021/6645373 (PMC8154280; doi:10.1155/2021/6645373)
Supplement: Supplementary Materials — Demographic, clinical, pathological, and biochemical data are available in the supplementary table. [file 6645373.f1.docx]

| **No.** | **Age** | **consanguinity** | **Sex** | **Pathological finding** | **Relapse** | **U.ptn/Cr . mg/mg** | **BUN** | **Cr.** | **T.Ptn.** | **Alb** | **Cholest** | **Serum C3** | **Serum C4** | **Mitochondrial complex I** |
| --- | --- | --- | --- | --- | --- | --- | --- | --- | --- | --- | --- | --- | --- | --- |
|  | **(yrs)** |  |  |  |  |  | **mg/dl** | **mg/dl** | **g/dl** | **g/dl** | **mg/dl** |  |  | **nmol/ml/min** |
| **SRNS** |  |  |  |  |  |  |  |  |  |  |  |  |  |  |
| 1 | 7 | positive | male | FSGS | 4 | 6.4 | 9 | 0.5 | 4 | 1.1 | 340 | 90.5 | 4.5 | 0.23 |
| 2 | 8 | positive | female | FSGS | 5 | 2.1 | 16 | 0.3 | 4.2 | 1.5 | 560 | 88 | 6..8 | 0.16 |
| 3 | 5 | positive | female | FSGS | 2 | 3.9 | 5 | 0.3 | 3.8 | 1.6 | 208 | 65.4 | 3.4 | 0.36 |
| 4 | 9 | positive | female | FSGS | 1 | 2.3 | 4 | 0.5 | 5.3 | 2.5 | 200 | 38.16 | 7.9 | 0.33 |
| 5 | 10 | positive | female | FSGS | 1 | 1.9 | 10 | 0.6 | 5.6 | 3 | 233 | 75 | 10 | 0.5 |
| 6 | 5 | positive | female | FSGS | 2 | 3.1 | 8 | 0.7 | 4.3 | 2 | 311 | 112 | 3.8 | 0.52 |
| 7 | 8 | positive | male | MCG | 5 | 3.3 | 15 | 0.4 | 5.2 | 1.7 | 283 | 98.5 | 4.9 | 0.49 |
| 8 | 7 | positive | male | FSGS | 4 | 3.2 | 8 | 0.5 | 4 | 1.1 | 500 | 55 | 7.5 | 0.29 |
| 9 | 9 | positive | male | FSGS | 3 | 3.2 | 11 | 0.4 | 5.2 | 2.5 | 308 | 46.9 | 10.2 | 0.3 |
| 10 | 12 | positive | male | MCG | 6 | 4.4 | 8 | 0.6 | 5 | 2.2 | 422 | 70.12 | 4.6 | 0.38 |
| 11 | 3 | negative | female | FSGS | 0 | 1.8 | 7 | 0.3 | 5.5 | 3 | 220 | 85.5 | 7.4 | 0.43 |
| 12 | 12 | positive | male | FSGS | 7 | 2.7 | 8 | 0.6 | 5.6 | 3.1 | 222 | 40 | 12.5 | 0.5 |
| 13 | 4 | negative | male | FSGS | 1 | 3.1 | 10 | 0.4 | 4.5 | 1.8 | 440 | 90.5 | 6.4 | 0.57 |
| 14 | 9 | positive | male | FSGS | 1 | 4.3 | 5 | 0.3 | 3.4 | 1.2 | 418 | 115 | 5.8 | 0.44 |
| 15 | 8 | positive | female | FSGS | 0 | 1.5 | 27 | 0.7 | 6 | 3.4 | 406 | 77.6 | 9.4 | 0.44 |
| 16 | 5 | positive | female | MCG | 1 | 3.2 | 10 | 0.4 | 4.8 | 1.6 | 427 | 45.4 | 3.45 | 0.4 |
| 17 | 4 | positive | female | IgA neph | 0 | 3.1 | 6 | 0.2 | 5.4 | 1.3 | 373 | 30.8 | 12.7 | 0.12 |
| 18 | 6 | positive | female | FSGS | 3 | 4.1 | 10 | 0.3 | 6.1 | 2 | 303 | 85.2 | 10.5 | 0.25 |
| 19 | 8 | positive | female | FSGS | 5 | 4.4 | 11 | 0.6 | 5.3 | 1.6 | 630 | 74.2 | 8.6 | 0.54 |
| 20 | 12 | positive | male | FSGS | 8 | 3.3 | 12 | 0.5 | 6.4 | 3.3 | 226 | 80 | 4.6 | 0.51 |
| 21 | 11 | positive | male | MCG | 1 | 3.1 | 10 | 0.3 | 5.1 | 1.6 | 324 | 95.4 | 3.9 | 0.109 |
| 22 | 12 | negative | female | FSGS | 7 | 5.8 | 12 | 0.5 | 5.1 | 1.8 | 364 | 76.19 | 7.8 | 0.147 |
| 23 | 3 | negative | male | NS | 4 | 1.5 | 13 | 0.7 | 7 | 3 | 270 | 102.3 | 10.45 | 0.278 |
| 24 | 7 | positive | male | FSGS | 5 | 2.9 | 5 | 0.3 | 4.2 | 1.4 | 460 | 90.7 | 2.7 | 0.026 |
| 25 | 10 | negative | female | FSGS | 5 | 3.3 | 9 | 0.4 | 5.5 | 1.9 | 464 | 65.3 | 5.6 | 0.102 |
| 26 | 4 | positive | male | FSGS | 3 | 1.1 | 17 | 0.6 | 6.6 | 3.1 | 227 | 72.2 | 4.7 | 0.252 |
| 27 | 7 | positive | female | FSGS | 4 | 4.6 | 19 | 0.7 | 4.6 | 2.1 | 400 | 45.5 | 4.4 | 0.198 |
| 28 | 12 | negative | female | MCG | 5 | 3.4 | 10 | 0.6 | 5.4 | 2.2 | 318 | 85.5 | 5.6 | 0.099 |
| 29 | 6 | positive | female | FSGS | 3 | 2.1 | 8 | 0.5 | 3.4 | 1.6 | 249 | 80.3 | 2.3 | 0.131 |
| 30 | 12 | positive | male | FSGS | 0 | 3.4 | 10 | 0.3 | 4 | 1.8 | 277 | 44.6 | 6.7 | 0.295 |
| 31 | 6 | positive | male | FSGS | 1 | 1.6 | 14 | 0.7 | 5.7 | 2.2 | 300 | 95.4 | 4.6 | 0.183 |
| 32 | 5 | negative | female | FSGS | 3 | 2.7 | 15 | 0.7 | 4.8 | 2.5 | 234 | 42.5 | 1.9 | 0.113 |
| 33 | 2.5 | positive | female | FSGS | 2 | 3.02 | 20 | 1.1 | 5.7 | 1.5 | 369 | 87.6 | 7.4 | 0.117 |
| 34 | 11 | negative | female | NS | 1 | 1.2 | 10 | 0.5 | 7.1 | 4 | 106 | 98.1 | 23.3 | 0.049 |
| 35 | 4 | positive | female | MCG | 3 | 2.8 | 12 | 0.5 | 6.8 | 3.8 | 265 | 102 | 22.6 | 0.025 |
| 36 | 6.5 | positive | male | FSGS | 5 | 3.5 | 11 | 0.3 | 4.2 | 1.3 | 516 | 75.6 | 12.9 | 0.074 |
| 37 | 5.5 | positive | male | FSGS | 4 | 4.6 | 18 | 0.7 | 5 | 2.1 | 497 | 77.2 | 20.4 | 0.067 |
| 38 | 5 | positive | female | FSGS | 2 | 2.2 | 19 | 0.6 | 6.4 | 3.4 | 350 | 64.2 | 11.6 | 0.183 |
| 39 | 2 | positive | female | FSGS | 1 | 3.2 | 10 | 0.3 | 4.7 | 1.8 | 277 | 82.6 | 8.2 | 0.094 |
| 40 | 12 | negative | female | FSGS | 5 | 2.4 | 35 | 2.2 | 5.9 | 3 | 192 | 118.4 | 19.3 | 0.085 |
| 41 | 6.5 | negative | male | NS | 6 | 5.12 | 18 | 0.6 | 5.2 | 2.5 | 377 | 100.6 | 25.7 | 0.043 |
| 42 | 10 | negative | male | NS | 8 | 2.9 | 12 | 0.7 | 5.1 | 2.4 | 300 | 94 | 3.3 | 0.133 |
| 43 | 7 | positive | female | MCG | 4 | 3.8 | 20 | 0.8 | 4.9 | 1.2 | 397 | 66.1 | 6.8 | 0.06 |
| 44 | 10 | positive | male | MCG | 5 | 3.6 | 30 | 1.9 | 5.6 | 1.6 | 365 | 57.3 | 4.1 | 0.072 |
| 45 | 8 | positive | female | FSGS | 7 | 4.7 | 25 | 1.2 | 4.9 | 1.7 | 448 | 49.8 | 11.2 | 0.022 |
| 46 | 7 | negative | male | FSGS | 6 | 5.8 | 24 | 1 | 5.2 | 1.3 | 358 | 80.2 | 20.3 | 0.067 |
| 47 | 7 | positive | female | FSGS | 5 | 3.8 | 11 | 0.6 | 5.3 | 2.2 | 302 | 106 | 16.3 | 0.076 |
| 48 | 12 | positive | male | FSGS | 4 | 4.03 | 15 | 0.9 | 6.5 | 2.8 | 380 | 83.6 | 14.8 | 0.2 |
| 49 | 12 | positive | male | FSGS | 8 | 6.7 | 38 | 2.8 | 5 | 1.5 | 528 | 74.2 | 9.7 | 0.35 |
| 50 | 4 | positive | female | FSGS | 3 | 4.7 | 9 | 0.3 | 4.3 | 0.6 | 417 | 65.3 | 10.2 | 0.36 |
| 51 | 2 | negative | female | FSGS | 2 | 5.54 | 6 | 0.2 | 3.2 | 1.04 | 307 | 60.2 | 11.7 | 0.64 |
| 52 | 4 | positive | male | FSGS | 5 | 7.68 | 32 | 2.3 | 3 | 1.1 | 412 | 54.9 | 20.2 | 0.56 |
| 53 | 3 | positive | female | FSGS | 4 | 6.1 | 30 | 1.7 | 3.3 | 1.2 | 593 | 101 | 23.6 | 0.63 |
| 54 | 11 | positive | female | NS | 6 | 5.7 | 29 | 1.7 | 3.9 | 1.9 | 466 | 114.6 | 10.9 | 0.47 |
| 55 | 12 | positive | male | NS | 8 | 6.03 | 40 | 2.5 | 3.9 | 1.1 | 452 | 78.6 | 4.6 | 0.44 |
| 56 | 5 | negative | male | FSGS | 4 | 4.1 | 21 | 0.9 | 2.8 | 0.8 | 582 | 88.5 | 5.2 | 0.39 |
| 57 | 8 | negative | male | FSGS | 6 | 5.15 | 34 | 2.4 | 3 | 1.4 | 604 | 64.2 | 2.1 | 0.25 |
| 58 | 10 | positive | female | FSGS | 5 | 3.96 | 20 | 1.4 | 4.3 | 1.6 | 651 | 85.6 | 4.5 | 0.56 |
| 59 | 11 | positive | male | FSGS | 7 | 5.9 | 16 | 0.8 | 3.8 | 1.5 | 405 | 98.3 | 4.4 | 0.64 |

|  |  |  |  |  |  |  |  |  |  |  |  |  |  |  |  |
| --- | --- | --- | --- | --- | --- | --- | --- | --- | --- | --- | --- | --- | --- | --- | --- |
|  |  |  |  |  |  |  |  |  |  |  |  |  |  |  |  |
| **SSNS** | |  |  |  |  |  |  |  |  |  |  |  |  |  |  |
| 60 | | 7 | negative | female | NS | 4 | 4.2 | 9 | 0.3 | 4.1 | 2 | 338 | 45.6 | 3.8 | 0.65 |
| 61 | | 5 | negative | male | FSGS | 4 | 5.4 | 19 | 0.9 | 4.5 | 1.9 | 210 | 64.3 | 1.4 | 0.64 |
| 62 | | 4 | negative | male | FSGS | 2 | 4.6 | 35 | 1.1 | 5.3 | 1.8 | 313 | 74.1 | 12.4 | 0.56 |
| 63 | | 7 | positive | female | FSGS | 4 | 6.1 | 30 | 1.2 | 6.2 | 2.2 | 512 | 87.6 | 3.2 | 0.41 |
| 64 | | 6 | negative | male | MCG | 3 | 4.3 | 28 | 0.8 | 4.4 | 1.2 | 423 | 103 | 2.6 | 0.62 |
| 65 | | 4 | negative | male | MCG | 5 | 7.9 | 40 | 2.2 | 5.9 | 1.8 | 321 | 100.6 | 8.9 | 0.67 |
| 66 | | 6.5 | negative | female | MCG | 4 | 5.7 | 34 | 1.02 | 4.6 | 3.1 | 420 | 114 | 14.6 | 0.46 |
| 67 | | 11 | positive | male | FSGS | 4 | 4.5 | 33 | 1.5 | 3.3 | 1.6 | 448 | 95.6 | 4.4 | 0.69 |
| 68 | | 8.5 | positive | male | FSGS | 3 | 4.23 | 28 | 0.8 | 4.7 | 2.2 | 357 | 78.4 | 2.7 | 0.66 |
| 69 | | 4 | negative | male | FSGS | 2 | 5.01 | 36 | 0.9 | 5.2 | 1.8 | 3.6 | 85.2 | 3.3 | 0.52 |
| 70 | | 6 | positive | female | FSGS | 0 | 6.03 | 30 | 1.3 | 6.1 | 1.6 | 412 | 112.3 | 2.7 | 0.42 |
| 71 | | 7.5 | negative | male | NS | 1 | 5.7 | 53 | 1.5 | 4.7 | 1.5 | 521 | 98.6 | 11.7 | 0.6 |
| 72 | | 9 | negative | female | FSGS | 4 | 5.5 | 35 | 2.1 | 3.9 | 0.9 | 550 | 76.5 | 2.8 | 0.64 |
| 73 | | 10 | positive | male | FSGS | 4 | 4.06 | 33 | 2.3 | 3.8 | 1.6 | 295 | 85.5 | 10.3 | 0.54 |
| 74 | | 10 | positive | male | FSGS | 3 | 6.2 | 40 | 1.4 | 2.1 | 1.7 | 319 | 97 | 12.7 | 0.37 |
| 75 | | 5 | positive | male | FSGS | 5 | 4.12 | 41 | 1.1 | 6.5 | 2.3 | 420 | 100 | 23.3 | 0.49 |
| 76 | | 4 | positive | female | NS | 2 | 5.5 | 38 | 2 | 5.9 | 2.2 | 404 | 120 | 4.5 | 0.62 |
| 77 | | 10 | negative | female | FSGS | 3 | 3.92 | 22 | 2.1 | 6.7 | 2.4 | 521 | 123 | 6.8 | 0.33 |
| 78 | | 6 | positive | male | FSGS | 2 | 3.3 | 28 | 1.5 | 5.4 | 3.1 | 321 | 145 | 7.4 | 0.53 |
| 79 | | 5 | negative | male | FSGS | 2 | 4.6 | 40 | 1.7 | 4.3 | 1.6 | 336 | 118 | 4.6 | 0.47 |
| 80 | | 8.5 | negative | male | MCG | 3 | 5.4 | 32 | 2.1 | 4.4 | 1.7 | 423 | 245 | 4.3 | 0.62 |
| 81 | | 10 | positive | female | MCG | 2 | 6.1 | 28 | 1.9 | 5.4 | 2.3 | 400 | 119 | 3.2 | 0.54 |
| 82 | | 11 | positive | male | MCG | 3 | 4.3 | 19 | 1.6 | 5.5 | 0.9 | 450 | 87 | 1.6 | 0.55 |
| 83 | | 8 | negative | male | FSGS | 2 | 2.7 | 22 | 2.3 | 4.6 | 1.1 | 403 | 95.6 | 16.4 | 0.36 |
| 84 | | 5 | positive | female | FSGS | 3 | 5.07 | 10 | 0.9 | 3.9 | 1.5 | 354 | 67.3 | 21 | 0.41 |
| 85 | | 5 | negative | male | FSGS | 1 | 5.51 | 17 | 0.9 | 3.6 | 2.5 | 445 | 112.2 | 17.3 | 0.35 |
| 86 | | 5 | positive | male | MCG | 3 | 7.3 | 23 | 1.2 | 3.3 | 3.1 | 345 | 124 | 5.1 | 0.46 |
| 87 | | 4 | negative | female | NS | 4 | 6.01 | 20 | 1.1 | 6.5 | 3.3 | 400 | 103 | 10.2 | 0.52 |
| 88 | | 6 | positive | male | NS | 2 | 6.6 | 19 | 1.3 | 6.6 | 1.9 | 252 | 120 | 11.6 | 0.61 |
| 89 | | 4 | positive | female | FSGS | 4 | 4.6 | 25 | 2.3 | 6.2 | 0.9 | 317 | 95.5 | 5.4 | 0.42 |
| 90 | | 5 | positive | male | FSGS | 1 | 5.2 | 23 | 2.2 | 4.9 | 0.8 | 330 | 88.6 | 5.5 | 0.53 |
| 91 | | 7 | positive | male | FSGS | 2 | 3.3 | 19 | 1.4 | 4.4 | 1.5 | 420 | 65.5 | 2.1 | 0.67 |
| 92 | | 8 | positive | male | MCG | 0 | 2.9 | 8 | 0.8 | 4.5 | 1.4 | 440 | 72.3 | 9.7 | 0.6 |
| 93 | | 10 | positive | male | MCG | 4 | 4.5 | 9 | 1.6 | 5.6 | 1.3 | 541 | 112 | 12.3 | 0.51 |
| 94 | | 7 | positive | male | FSGS | 3 | 4.4 | 10 | 1.3 | 5.6 | 2.3 | 368 | 206 | 6.9 | 0.52 |
| 95 | | 10 | positive | female | FSGS | 3 | 4.6 | 14 | 0.8 | 5.5 | 2.2 | 345 | 149 | 6.6 | 0.32 |
| 96 | | 6 | positive | male | FSGS | 1 | 5.5 | 16 | 0.4 | 4.6 | 2.4 | 465 | 108 | 5.4 | 0.43 |
| 97 | | 6 | negative | Female | FSGS | 0 | 4.9 | 23 | 1.3 | 4.4 | 1.9 | 420 | 87.4 | 1.2 | 0.62 |
| 98 | | 4 | negative | Male | FSGS | 2 | 4.4 | 22 | 0.6 | 4.6 | 1.8 | 440 | 76.3 | 9.7 | 0.41 |
| 99 | | 10 | negative | Male | FSGS | 2 | 3.65 | 20 | 0.9 | 6.5 | 2.3 | 354 | 45.2 | 22.3 | 0.23 |
| 100 | | 8 | positive | female | FSGS | 3 | 7.21 | 17 | 0.8 | 6.6 | 2.2 | 336 | 103 | 11.3 | 0.32 |
| 101 | | 3 | positive | female | FSGS | 4 | 4.3 | 8 | 1.3 | 6.5 | 3.6 | 100 | 114 | 10.2 | 0.42 |
| 102 | | 7 | negative | Male | FSGS | 0 | 2.4 | 10 | 1.1 | 6.6 | 2.9 | 341 | 64.2 | 6.5 | 0.28 |
| 103 | | 6 | positive | female | MCG | 1 | 5.02 | 15 | 0.6 | 6.4 | 3.5 | 199 | 67.8 | 7.4 | 0.36 |
| 104 | | 4 | positive | Male | MCG | 1 | 1.8 | 13 | 0.8 | 7 | 3.2 | 250 | 65.3 | 7.7 | 0.42 |
| 105 | | 6 | positive | Male | MCG | 2 | 2.9 | 12 | 1.2 | 6.9 | 3.3 | 222 | 45.9 | 10.2 | 0.37 |
| 106 | | 2.8 | negative | female | FSGS | 0 | 3.3 | 12 | 0.7 | 6.3 | 3 | 175 | 100.7 | 5.6 | 0.42 |
| 107 | | 3 | positive | Male | FSGS | 0 | 4.4 | 19 | 0.6 | 6.5 | 2.1 | 194 | 82.6 | 6.3 | 0.52 |
| 108 | | 15 | positive | female | FSGS | 3 | 2.9 | 10 | 0.5 | 5.8 | 2.9 | 207 | 79.4 | 2.2 | 0.67 |
| 109 | | 5 | negative | Male | FSGS | 1 | 3.2 | 8 | 0.9 | 6.3 | 2.6 | 180 | 80.2 | 9.4 | 0.36 |
| 110 | | 2 | positive | Male | FSGS | 0 | 4.5 | 12 | 1.2 | 6.2 | 2.2 | 360 | 106 | 5.5 | 0.29 |
| 111 | | 4 | negative | female | MCG | 1 | 3.07 | 17 | 0.9 | 7.1 | 3.1 | 240 | 64.2 | 1.6 | 0.42 |
| 112 | | 3 | negative | Male | MCG | 1 | 2.8 | 13 | 0.7 | 7.6 | 3.3 | 330 | 75.5 | 2.3 | 0.33 |
| 113 | | 13 | positive | Male | MCG | 2 | 4.6 | 10 | 0.6 | 6.9 | 3.5 | 315 | 70.9 | 3.3 | 0.54 |
| 114 | | 2 | negative | female | MCG | 0 | 4.1 | 11 | 0.9 | 6.9 | 2.9 | 211 | 103 | 2.6 | 0.23 |
| 115 | | 1.5 | negative | female | FSGS | 0 | 5.3 | 16 | 0.8 | 5.4 | 2.2 | 305 | 100 | 4.6 | 0.34 |
| 116 | | 3 | positive | Male | FSGS | 1 | 3.6 | 18 | 1 | 6.2 | 2.9 | 293 | 85.5 | 4.4 | 0.35 |
| 117 | | 7 | positive | Male | FSGS | 1 | 4.2 | 13 | 1.4 | 7.5 | 2.4 | 273 | 64.9 | 1.7 | 0.21 |
| 118 | | 10 | negative | Male | FSGS | 2 | 3.9 | 6 | 1 | 7.1 | 2.8 | 165 | 75 | 1.3 | 0.32 |
| 119 | | 3.5 | negative | female | FSGS | 0 | 4.5 | 8 | 0.9 | 6.3 | 1.8 | 218 | 77.8 | 1.2 | 0.51 |
| 120 | | 8 | negative | male | FSGS | 1 | 3.6 | 10 | 0.6 | 6.4 | 1.9 | 230 | 63.3 | 2.6 | 0.62 |

| **FSGS** | Focal segmental glomerulosclerosis | | |  | **SRNS** | Steroid resistant nephrotic syndrome | | | |
| --- | --- | --- | --- | --- | --- | --- | --- | --- | --- |
| **MCGN** | Minimal changes glomerulonephritis | | |  | **SSNS** | steroid sensitive nephrotic syndrome | | | |
| **NS** | nonspecific changes | |  |  |  |  |  |  |  |
| **IgA neph** | IgA nephropathy |  |  |  |  |  |  |  |  |
